# The Intratumor Microbiota Signatures Associate With Subtype, Tumor Stage, and Survival Status of Esophageal Carcinoma

**DOI:** 10.3389/fonc.2021.754788

**Published:** 2021-10-27

**Authors:** Yangyang Wang, Hua Guo, Xiaoguang Gao, Jihan Wang

**Affiliations:** ^1^ School of Electronics and Information, Northwestern Polytechnical University, Xi’an, China; ^2^ Department of Nursing, Shaanxi Provincial People’s Hospital, Xi’an, China; ^3^ Institute of Medical Research, Northwestern Polytechnical University, Xi’an, China

**Keywords:** esophageal carcinoma, intratumor microbiota, The Cancer Microbiome Atlas, The Cancer Genome Atlas, microbial biomarker

## Abstract

Altered human microbiome characteristic has been linked with esophageal carcinoma (ESCA), analysis of microbial profiling directly derived from ESCA tumor tissue is beneficial for studying the microbial functions in tumorigenesis and development of ESCA. In this study, we identified the intratumor microbiome signature and investigated the correlation between microbes and clinical characteristics of patients with ESCA, on the basis of data and information obtained from The Cancer Microbiome Atlas (TCMA) and The Cancer Genome Atlas (TCGA) databases. A total of 82 samples were analyzed for microbial composition at various taxonomic levels, including 40 tumor samples of esophageal squamous cell carcinoma (ESCC), 20 tumor samples of esophageal adenocarcinoma (EAD), and 22 adjacent normal samples. The results showed that the relative abundance of several microbes changed in tumors compared to their paired normal tissues, such as *Firmicutes* increased significantly while *Proteobacteria* decreased in tumor samples. We also identified a microbial signature composed of ten microbes that may help in the classification of ESCC and EAD, the two subtypes of ESCA. Correlation analysis demonstrated that compositions of microbes *Fusobacteria*/*Fusobacteriia*/*Fusobacteriales*, *Lactobacillales*/*Lactobacillaceae*/*Lactobacillus*, *Clostridia*/*Clostridiales*, *Proteobacteria*, and *Negativicutes* were correlated with the clinical characteristics of ESCA patients. In summary, this study supports the feasibility of detecting intratumor microbial composition derived from tumor sequencing data, and it provides novel insights into the roles of microbiota in tumors. Ultimately, as the second genome of human body, microbiome signature analysis may help to add more information to the blueprint of human biology.

## Introduction

Esophageal carcinoma (ESCA) is a common type of cancer and one of the leading causes of mortality associated with the gastrointestinal tract. There are two main histological subtypes of ESCA, esophageal squamous cell carcinoma (ESCC) and esophageal adenocarcinoma (EAD). The two subtypes differ significantly in incidence, geographic distribution, and etiology. ESCC accounts for almost 90% of the ESCA incidence each year, and the geographic distribution of ESCC varies greatly, with the highest incidence rates occurring in Asia, especially China. Approximately half of all ESCC cases worldwide is reported in China, and these high rates are mainly due to China’s large population (1). In the West, EAD represents the main histological subtype and its incidence has increased rapidly over the past 30 years (2). Although the prognosis of EAD has slightly improved over the last few decades, it is still worse than that of most other cancer types. Moreover, since most patients are diagnosed at late stages, the motility of esophageal carcinoma remains high; in most countries, approximately only 15%-25% of patients survive 5 years.

The etiology of ESCA is multifactorial and includes cigarette smoking, alcohol consumption, and low fruit/vegetable intake for ESCC and gastroesophageal reflux disease (GERD), Barrett’s esophagus (BE), obesity, low fruit/vegetable intake, and cigarette smoking for EAD. The current understanding of these risk factors cannot fully explain the etiology of ESCC and EAD. Microbiota have recently emerged as novel tumorigenesis regulators and biomarkers in disease and multiple types of cancer, including ESCA (3–6). Microbial dysbiosis contributes to cancer susceptibility through complex mechanisms, including inducing inflammation and immune disfunction and interfering with anticancer drug pharmacodynamics. Dysbiosis of the gut microbiota (GM) has been studied in ESCA patients (3, 7). In addition, investigation of the esophageal microbiota is a relatively new approach in the field of ESCA (8). Several studies have indicated alterations in the esophageal microbiota in esophagitis, BE, EAD, and ESCC (7, 9, 10). There is evidence that the microbial composition of the esophagus is diverse, with gram-positive bacteria dominating in healthy conditions, while gram-negative bacteria predominating in disease status including GERD and BE (8). Exploring esophageal microbiota changes will help us better understand the tumor pathophysiology and provide potential diagnostic and/or therapeutic approaches for ESCA.

Recently, The Cancer Microbiome Atlas (TCMA) revealed a pan-cancer analysis identifying tissue-resident microbiota (11). The sample types that were analyzed for microbial prevalence were derived from The Cancer Genome Atlas (TCGA) program, and over 20,000 primary cancer and matched normal samples spanning 33 cancer types were molecularly characterized. Until now, the TCMA has been a resource for exploring the tissue-resident microbiota prevalence in several cancer types, including tissues of the oropharynx, esophagus, stomach, and colorectum. Previously, we explored the microbiota signature in four major types of gastrointestinal cancer, and the results demonstrated that the microbial profile is highly site-specific and notably differed between upper and lower gastrointestinal tumors (12). Several other studies have also investigated the intratumor microbiota derived from TCGA sequences of different cancer types (13, 14). In the study of a TCGA breast cancer cohort, the results indicated an increased *Proteobacteria* presence in tumor tissues, while the composition of *Actinobacteria* was elevated in the adjacent normal tissues (15). Rodriguez et al. detected the global microbial composition in tumor and adjacent normal tissues across 9 TCGA cancer cohorts (16). Microbiome analysis from tumor tissues as well as human blood samples will also reveal a new class of microbial-based cancer diagnostics (17). Overall, exploring the intratumor microbial signature will help improve our knowledge of the host-microbiota interaction, which is important to understand the linkage of dysbiosis with chronic inflammation and processes that influence tumorigenesis.

Understanding the relationship between clinical phenotype information and multiomics data such as the genome or microbiome is critical for human biological and medical research. To the best of our knowledge, no studies have been conducted to investigate the comprehensive microbial signature or its relationship with the clinicopathological characterization of ESCA. Here, we profiled the microbiome of patients with ESCA from the TCMA, and also analyzed the clinical phenotype and survival data of the corresponding samples from the TCGA. The global microbial composition at the phylum, class, order, family, and genus levels of tumor and noncancerous adjacent normal tissues was calculated to analyze the differential microbes. We further evaluated the correlation between the microbes and the clinical variables of the tumors. Specifically, we identified the microbial signatures related to cancer subtype, tumor stage, and survival status. We believe that the intratumor microbial study will provide a better understanding of dysbiosis and establish a new foundation for studying host-microbiota interactions and the role of microbiota in the tumorigenesis of esophageal carcinoma.

## Materials and Methods

### Sample Acquisition and Information Collection

**Figure 1:**
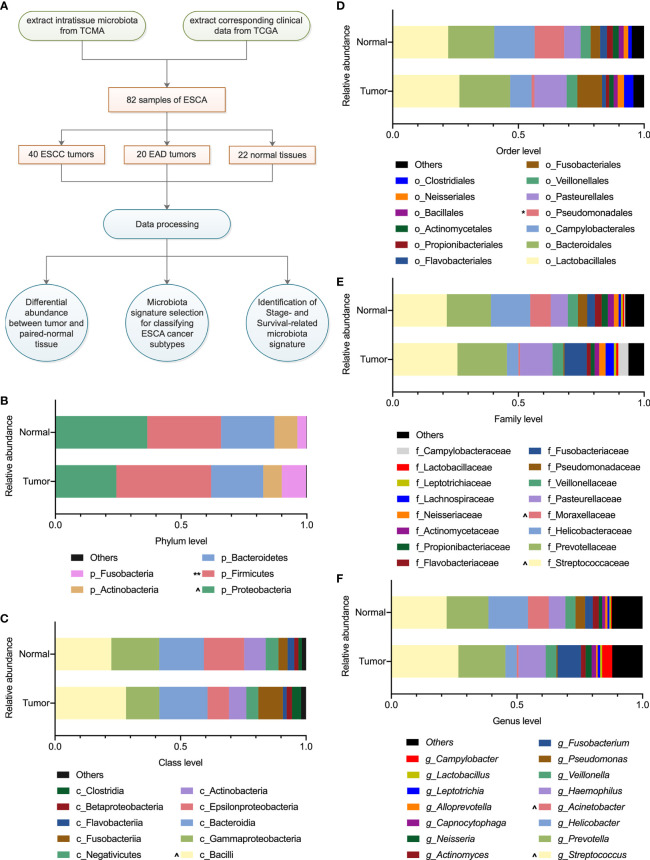
Overview of the study design and microbiota profiling. **(A)** Schematic overview of the study design. TCMA, the cancer microbiome atlas; TCGA, the cancer genome atlas; ESCA, esophageal carcinoma; ESCC, esophageal squamous cell carcinoma; EAD, esophageal adenocarcinoma. **(B–F)** The composition of microbiota with an average abundance > 1% in tumor and paired normal tissues at the phylum, class, order, family, and genus levels, respectively. The relative microbial abundance in tumors compared with normal samples, ^0.1 < *P* < 0.01, *P < 0.05, **P < 0.01 in paired-samples *t*-test.

In this study, the microbiota profiles of samples from 82 cases (including 40 ESCC tumors, 20 EAD tumors, and 22 noncancerous adjacent tissues used as normal samples) at the phylum, class, order, family, and genus levels were obtained from the TCMA database (https://tcma.pratt.duke.edu/); the corresponding clinical phenotype information and survival data for the 82 patients were obtained from the TCGA program (for phenotype information, https://gdc-hub.s3.us-east-1.amazonaws.com/download/TCGA-ESCA.GDC_phenotype.tsv.gz; for survival data, https://gdc-hub.s3.us-east-1.amazonaws.com/download/TCGA-ESCA.survival.tsv). **Figure 1A** displays an overview of the study design, and the clinical information about the patient samples is summarized in **Table 1**.

**Table 1:** Clinical characteristics of cases in this study (information derived from the TCGA database).

Clinical characteristics		ESCA tumor tissue		Normal tissue
		ESCC	EAD	
Age (year)		36-90	47-86	51-90
[range (mean ± SD)]		(59.45 ± 10.60)	(72.75 ± 10.75)	(73.45 ± 10.98)
Gender	Male	36	15	15
	Female	4	5	7
Pathologic T-stage	T1	2	8	–
	T2	10	2	–
	T3	24	9	–
	T4	2	0	–
	Not reported	2	1	–
Pathologic N-stage	N0	27	6	–
	N1	11	8	–
	N2	0	3	–
	N3	0	2	–
	Not reported	2	1	–
Pathologic M-stage	M0	35	9	–
	M1	1	1	–
	MX	1	6	–
	Not reported	3	4	–
Overall stage	Stage I	2	6	–
	Stage II	26	4	–
	Stage III	9	6	–
	Stage IV	1	1	–
	Not reported	2	3	–
Overall survival status	Alive	28	14	–
	Dead	11	6	–
	Not reported	1	0	–
Overall survival time (day)		96-1688	9-1458	–
[range (mean ± SD)]		(551.90 ± 435.05)	(405.28 ± 284.02)	
**Total number**		**40**	**20**	**22**

ESCA, esophageal carcinoma; EAD, esophageal adenocarcinoma; ESCC, esophageal squamous cell carcinoma. SD, standard deviation.

### Microbiota Abundance Analysis of Tumor and Normal Tissues of Esophageal Carcinoma

The relative abundance of the microbiota composition at the phylum, class, order, family, and genus levels of each sample was calculated, and the microbial composition with an average relative abundance > 1% was selected for further analysis. In this study, there were 22 pairs of strictly matched tumor-adjacent normal samples of ESCA. The paired-samples *t*-test was used to analyze the differential microbial composition in tumors and their normal tissues, with a false discovery rate (FDR)-adjusted *P*-value < 0.05 considered significant. In addition, linear discriminant analysis effect size (LEfSe) analysis was performed by using OECloud tools at https://cloud.oebiotech.cn. Specifically, the nonparametric factorial Kruskal-Wallis (KW) sum-rank test and Wilcoxon rank-sum test were used to identify taxa biomarkers for tumor and normal samples, and linear discriminant analysis (LDA) was further performed to evaluate the microbial effects for each group. The microbes with LDA values > 2 and *P* < 0.1 were considered significantly enriched in that group.

### Microbiota Signature Selection for Classifying Subtypes of Esophageal Carcinoma

There were 40 ESCC and 20 EAD tumor samples in the esophageal carcinoma group. We investigated whether these two cancer subtypes could be classified based on the tumor microbiota profile. The SHapley Additive exPlanations (SHAP) (18, 19) theoretic approach was performed for microbial feature selection to identify the more important microbial profile, which may predict the classification of the two different cancer subtypes. The global microbiota (as the variables to distinguish the two cancer subtypes) importance scores were evaluated and visualized by SHAP, and we then selected the top ten most important microbial features for further analysis. Principal component analysis (PCA) and partial least squares discrimination analysis (PLS-DA) were performed by using the packages “FactoMineR” and “mixOmics” in R version 4.0.2, respectively.

### Identification of Stage- and Survival-related Microbiota Signatures for Esophageal Carcinoma

The microbiota profile of ESCA at the phylum, class, order, family, and genus levels derived from the TCMA database and the corresponding clinical information of all the samples obtained from the TCGA database were integrated for correlation analysis. Specifically, the Pearson cor.test () function in R version 4.0.2 was performed to analyze the correlation between the relative abundance of specific microbiota and tumor stage. Kaplan‐Meier survival analysis was performed to assess the survival-related microbiota. The “survival” and “survminer” packages in R version 4.0.2 were used for survival analysis and curve visualization based on the microbial composition.

## Results

### Differential Microbiota Signatures in Tumor and Normal Tissues of Esophageal Carcinoma

There were 82 samples of esophageal carcinoma (**Table 1**) in the TCMA database. A total of 11, 22, 38, 67, and 221 taxa were obtained for each sample at the phylum, class, order, family, and genus levels, respectively. **Table S1** summarizes the global microbial profiling at each taxonomic level. We then explored the differential microbial compositions between tumor and normal tissues. Overall, there were 5, 10, 13, 16, and 15 microbial taxa with an average relative abundance > 1% at the phylum, class, order, family, and genus levels, respectively (**Figures 1B–F**). At the phylum level, the intratissue microbiota was dominated by *Proteobacteria* (36.5% for normal tissue, 24.2% for tumor tissue) and *Firmicutes* (29.3% for normal tissue, 37.7% for tumor tissue), followed by *Bacteroidetes* (21.2% for normal tissue, 20.7% for tumor tissue), *Actinobacteria* (9.2% for normal tissue, 7.4% for tumor tissue), and *Fusobacteria* (3.6% for normal tissue, 9.7% for tumor tissue). The microbial composition of *Firmicutes* increased significantly (*P* < 0.01), while that of *Proteobacteria* decreased (0.1 < *P* < 0.05) in tumor samples compared with their paired normal tissues (**Figure 1B**). As **Figures 1C–F** show, the difference in microbial composition profiling in tumors was not obviously significant compared with that in normal tissues, except that the relative abundance of *Pseudomonadales* was less abundant in tumors than in normal samples at the order level (*P* < 0.05, **Figure 1D**).

**Figure 2:**
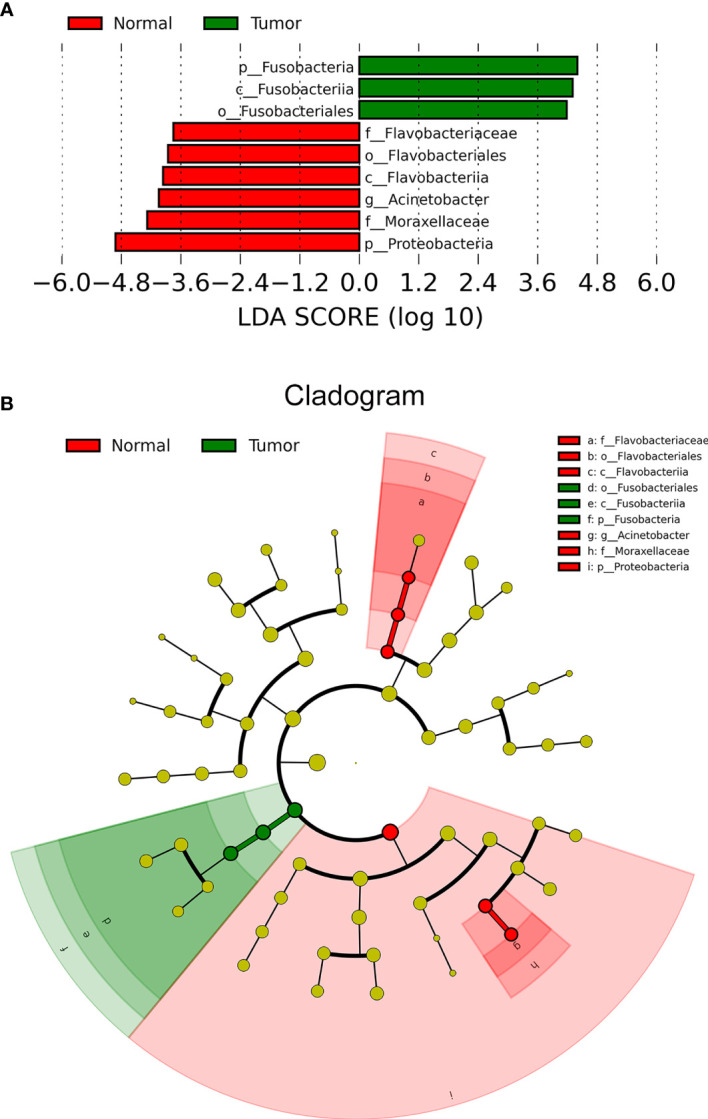
LEfSe analysis identifying tumor- and normal-enriched microbiota. **(A)** The LDA score of specific microbial taxa in the tumor group and normal group. Threshold LDA score is 2. **(B)** Cladogram showing tumor- and normal-enriched microbial taxa. LEfSe, linear discriminant analysis effect size; LDA, linear discriminant analysis.

LEfSe analysis helps to identify specific enriched microbial biomarkers for different groups. As **Figure 2** shows, the compositional abundances of *Fusobacteria*/*Fusobacteriia*/*Fusobacteriales* were higher in tumor tissues, while compositional abundances of *Proteobacteria*, *Moraxellaceae*, *Acinetobacter*, and *Flavobacteriia*/*Flavobacteriales*/*Flavobacteriaceae* were enriched in normal tissues. Specifically, the compositional positive ratio of order *Fusobacteriales* was 63.6% (14 of 22) for tumor and 68.2% (15 of 22) for normal tissue, while the average abundance of *Fusobacteriales* for the positive samples was higher (0.1 < *P* < 0.05) in tumor (14 positive samples with an average abundance of 15.4%) than that in normal tissues (15 positive samples with an average abundance of 5.6%). In contrast, a higher compositional positive ratio of *Flavobacteriaceae* (5 of 22 for tumor vs. 12 of 22 for normal, *P* < 0.05) and *Moraxellaceae* (2 of 22 for tumor vs. 6 of 22 for normal, *P* < 0.01) at the family level as well as *Acinetobacter* (2 of 22 for tumor vs. 6 of 22 for normal, *P* < 0.01) at the genus level was detected more in normal tissues than in tumors.

### Identification of Intratumor Microbiota Signatures Associated With Cancer Subtypes of Esophageal Carcinoma

**Figure 3:**
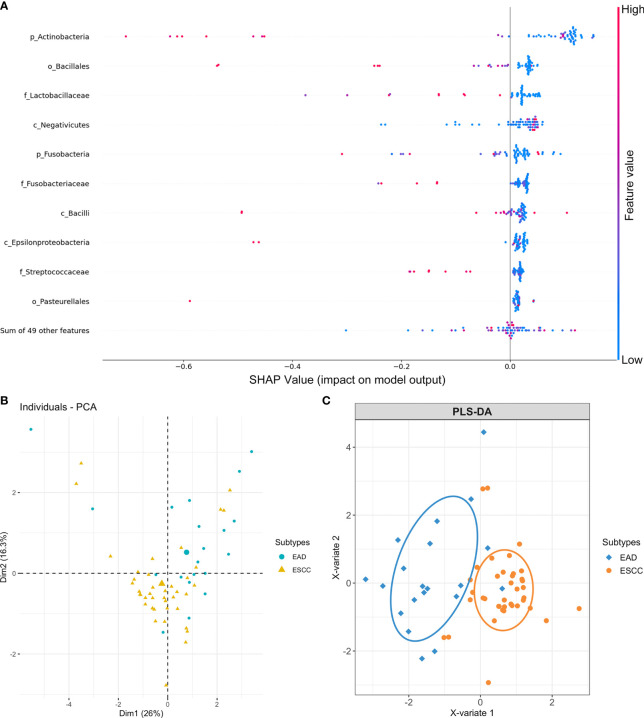
Microbial feature selection in predicting different cancer subtypes of esophageal carcinoma. **(A)** Summary plots of the importance values of the top 10 predictable microbes. **(B)** PCA plots and **(C)** PLS-DA plots based on the top 10 microbial features display the EAD and ESCC subtypes of esophageal carcinoma.

As the ESCA tumor samples were histologically subdivided into ESCC and EAD subtypes, we further investigated whether the intratumor microbial signature was somehow subtype-correlated in esophageal carcinoma. The SHAP (Shapley additive explanations) approach was applied to select the most valuable features in predicting the different groups, as it provided reference information about feature ranking and feature selection. Previously, a total of 59 microbial taxa were identified with an average relative composition > 1% at different taxonomic levels. To prioritize microbes of the 59 taxa, we relied on feature importance (contribution) obtained from SHAP to evaluate the whole importance value of each individual microbe in predicting cancer subtypes. A signature containing the top 10 microbial features was identified from the 59 microbial taxa as predictable factors, including *Actinobacteria*, *Fusobacteria*, *Bacilli*, *Epsilonproteobacteria*, *Negativicutes*, *Bacillales*, *Pasteurellales*, *Fusobacteriaceae*, *Lactobacillaceae*, and *Streptococcaceae* (**Figure 3A**). PCA (**Figure S1A**) and PLS-DA (**Figure S1B**) of all 59 microbial profiles were performed before feature ranking and selection. A relatively improved separate model was observed when performing PCA (**Figure 3B**) and PLS-DA (**Figure 3C**) based on the ten microbial features obtained from SHAP after feature ranking and selection.

### Abundances of Specific Microbes in Relation to Patients’ Clinical Characteristics

**Figure 4:**
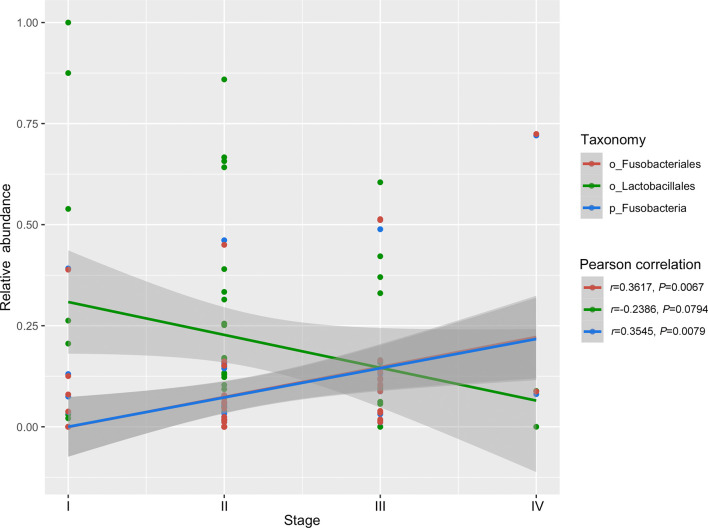
Correlation of specific microbes with the tumor stage status of ESCA. The analysis was performed using Pearson correlation in R.

**Figure 5:**
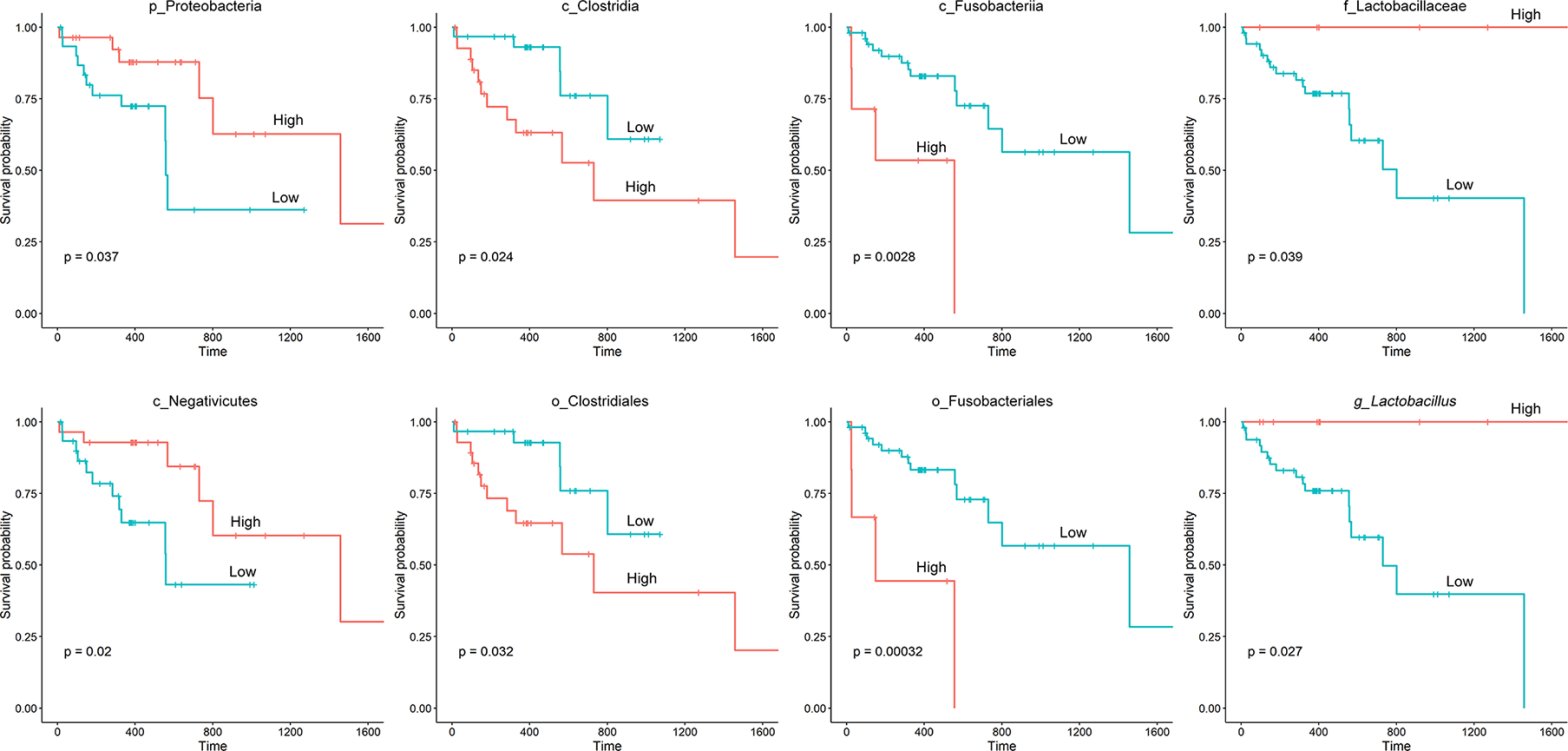
Kaplan‐Meier survival curves based on microbial composition. The “survival” and “survminer” packages in R were used for survival analysis. The process included determining the optimal cut-off point of variables, categorizing variables into “high” and “low” subgroups based on microbial abundance, fitting survival curves and visualization.

The TCGA database contains comprehensive clinical characterization of multiple types of cancer. **Table 1** summarizes the clinicopathological information of the 82 cases analysed in this study. We next investigated whether there were specific microbes associated with the clinicopathological variables of ESCA patients. The results showed that the composition of *Fusobacteria*/*Fusobacteriales* was positively correlated (*P* < 0.01), while the relative abundance of *Lactobacillales* was negatively correlated (0.05 < *P* < 0.1) with the tumor stage status of ESCA (**Figure 4**). The survival analysis indicated that the enrichment of several microbes could reflect the overall survival probability of patients (**Figure 5**). Detailed information about the survival status of tumor patients and the microbial composition were summarized in **Table S2**. High abundances of *Proteobacteria*, *Negativicutes*, and *Lactobacillaceae*/*Lactobacillus* were associated with better prognosis (*P* < 0.05), while a high composition of *Clostridia*/*Clostridiales* and *Fusobacteriia*/*Fusobacteriales* reflected poorer prognosis (*P* < 0.05). The eight microbes were then applied in a multivariate Cox regression analysis. Four microbes were identified as independent prognostic factors of ESCA patients (*P* < 0.05), as shown in **Table 2**.

**Table 2:** Multivariate Cox regression analysis of the independent significance of eight microbes as prognostic factors.

Microbes		HR	HR (0.95 lower)	HR (0.95 upper)	*P*-value
Phylum	*Proteobacteria*	5.61E-02	1.27E-03	2.48E+00	0.136
Class	*Clostridia*	Inf	Inf	Inf	**<2E-16**
	*Fusobacteriia*	3.94E-86	3.47E-87	4.48E-85	**<2E-16**
	*Negativicutes*	7.34E-03	7.57E-07	7.11E+01	0.294
Order	*Clostridiales*	0.00E+00	0.00E+00	0.00E+00	**<2E-16**
	*Fusobacteriales*	1.11E+86	9.81E+84	1.27E+87	**<2E-16**
Family	*Lactobacillaceae*	0.00E+00	0.00E+00	Inf	0.988
Genus	*Lactobacillus*	1.60E+298	0.00E+00	Inf	0.994

HR, hazard ratio. The results were analyzed by multivariate Cox regression model based on “survival” package in R.

There were four microbes with P < 0.05 in the multivariate Cox regression analysis, as showed in bold values.

It is essential to assess clinical-pathological prognostic factors such as lymph nodes, esophageal wall size and infiltration, and metastasis in relation to the microbiota under investigation. The information about lymph nodes in the current study was relatively incomplete (**Table S3**). As a result, we did not conduct the lymph node microbiota correlation analysis, which will require more attention in future research.

## Discussion

Studies on the bacterial or viral composition of human tumors using sequencing data from databases such as the TCGA have recently emerged (14, 20). Investigation of the intratumor microbiota will provide valuable information for better understanding the occurrence and progression of tumors. In addition to recent studies about microbiota changes in esophageal disease, our research performed a more comprehensive investigation of microbial characteristics in ESCA.

After microbial detection and calculation at different taxonomic levels, we found differential microbial abundance in tumor and normal samples of ESCA. Consistent with other studies, the relative abundance of *Firmicutes* and *Fusobacteria* increased, while that of *Proteobacteria* decreased in esophageal tumors compared with normal tissues (21–23). In another study, the enriched composition of *Firmicutes* and the unenriched composition of *Proteobacteria* were reported to be associated with BE (24). The LEfSe analysis in this study also indicated *Fusobacteria*/*Fusobacteriia*/*Fusobacteriales* as tumor-enriched microbes, suggesting that it might be a potential biomarker for the tumorigenesis and development of ESCA. In general, the alteration of microbial abundance at the class, order, family, and genus levels in tumors compared with adjacent normal tissue was not significant.

The human genome has been referred to as the blueprint of human biology (25). It is well established that cancer genome signature analysis helps to predict different cancer systems and their subtypes and contributes to precision medicine (26–28). The microbiome, as the second genome of the human body, plays crucial roles in health and disease (25, 29). A study reported that the intracellular microbiome of human tumors is tumor type-specific across multiple types of tumors, and intratumor bacteria or their predicted functions correlate with tumor types, subtypes, patient smoking status, and the response to immunotherapy (30). The microbiome could also be a potential biomarker/rule for subgrouping different cancer subtypes and used as a factor for exploring the complicated microenvironment components associated with tumorigenesis (31). In our research, we further identified a signature containing 10 microbial features that was somehow predictive of ESCC and EAD, the two subtypes of ESCA, by applying the SHAP approach. The human oral cavity harbours the second most abundant microbiota after the gut microbiota in the gastrointestinal tract (32). Microorganisms that exist in the oral cavity and its contiguous extensions (stopping at the distal esophagus) are all considered the oral microbiome and are altered within different oral structures and tissues (33). Here, we provide evidence that the microbial signature could be cancer subtype-related.

We then examined the relationship between specific microbes and the clinical index of ESCA patients by integrating the TCMA microbiome profile with clinical data from the TCGA. There were few links between tumor stage and microbial abundance, except that the composition of tumor-enriched *Fusobacteria*/*Fusobacteriales* was found to be positively correlated with tumor stage. *Fusobacteria* contribute to the formation of a proinflammatory microenvironment that promotes the colorectal neoplasia progression by recruiting tumor-infiltrating immune cells (34). In addition to its role in in colorectal cancer, *Fusobacteria* has been reported to be enriched in various cancer types, including oral, stomach, and breast cancer (35, 36). Studies have demonstrated that breast cancer colonized by *Fusobacterium nucleatum* accelerates tumor growth and metastasis (36). Furthermore, the high relative abundance of *Fusobacteriia*/*Fusobacteriales* correlated with a poorer prognosis in ESCA patients in our study, indicating that targeting *Fusobacteria* may be beneficial for the treatment of not only colorectal cancer but also other types of cancers. Thus, exploring the intratumor microbiome signature will facilitate the discovery of novel microbial biomarkers for cancer research.

## Conclusion

In this study, we conducted a comprehensive analysis of the intratumor microbiome in ESCA samples. Taken together, there are differences in the abundance of several microbial taxa between the tumor and adjacent normal tissues, and the potential functions of these microbes in ESCA merit further study. We also identified the intratumor microbiota signatures that were correlated with the subtype, tumor stage, and survival status of ESCA. We expect that our research will facilitate a better understanding of the intratumor microbiome of ESCA and identify potential biomarkers for the disease, as well as provide a novel perspective on the role of the microbiome in tumors, since studies of genome variation and disease risk will necessitate the integration of human and microbial genomic data.

Our study has limitations; the number of tumor and paired normal tissue samples in the subgroups was relatively small and did not allow us to make any generalizable conclusions. Large-scale and mechanistic studies are needed to further confirm the results of this study.

## Data Availability Statement

The original contributions presented in the study are included in the article/**Supplementary Material**. Further inquiries can be directed to the corresponding author.

## Author Contributions

YW and JW designed, performed the study, and co-wrote the manuscript. HG and XG contributed to data and statistical analysis, and reviewed the manuscript. All authors contributed to the article and approved the submitted version.

## Conflict of Interest

The authors declare that the research was conducted in the absence of any commercial or financial relationships that could be construed as a potential conflict of interest.

## Publisher’s Note

All claims expressed in this article are solely those of the authors and do not necessarily represent those of their affiliated organizations, or those of the publisher, the editors and the reviewers. Any product that may be evaluated in this article, or claim that may be made by its manufacturer, is not guaranteed or endorsed by the publisher.

## Supplementary Material

Supplementary Figure 1 PCA and PLS-DA plots based on the 59 microbial taxa displaying EAD and ESCC subtypes of esophageal carcinoma. **(A)** PCA plots. **(B)** PLS-DA plots.
