# Supplementary figures and images for: The Intratumor Microbiota Signatures Associate With Subtype, Tumor Stage, and Survival Status of Esophageal Carcinoma
Source: Front Oncol. 2021 Oct 27;11:754788. doi: 10.3389/fonc.2021.754788 (PMC8578860; doi:10.3389/fonc.2021.754788)

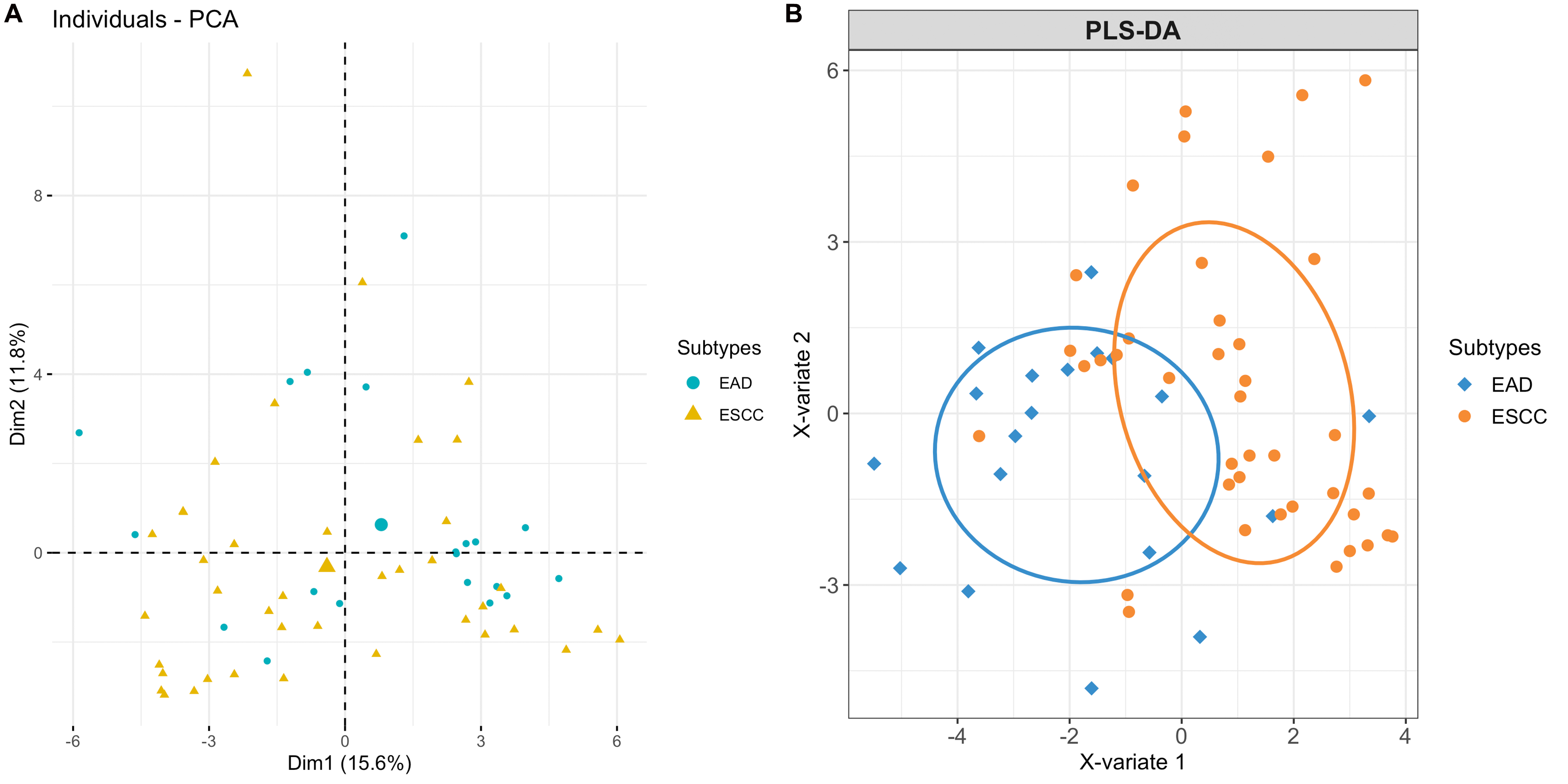

Supplement: Supplementary Figure 1 — PCA and PLS-DA plots based on the 59 microbial taxa displaying EAD and ESCC subtypes of esophageal carcinoma. (A) PCA plots. (B) PLS-DA plots. [file Image_1.tif]
